# Psychometric property of the Japanese version of self-efficacy for managing chronic disease scale in individuals with chronic diseases

**DOI:** 10.1016/j.heliyon.2024.e40218

**Published:** 2024-11-09

**Authors:** Megumi Hazumi, Mayumi Kataoka, Ayako Nakashita, Kentaro Usuda, Michi Miyake, Chiaki Kamikawa, Daisuke Nishi, Naoaki Kuroda

**Affiliations:** a Department of Public Mental Health Research, National Institute of Mental Health, National Center of Neurology and Psychiatry, 4-1-1 Ogawahigashicho, Kodaira, Tokyo, 187-8553, Japan; b Department of Sleep-Wake Disorders, National Institute of Mental Health, National Center of Neurology and Psychiatry, 4-1-1 Ogawahigashicho, Kodaira, Tokyo, 187-8553, Japan; c Department of Mental Health, Graduate School of Medicine, The University of Tokyo, 7-3-1 Hongo, Bunkyo-ku, Tokyo, 113-0033, Japan; d Human Developmental Sciences, Humanities and Sciences, Ochanomizu University, 2-1-1 Otsuka, Bunkyo-ku, Tokyo, 112-8610, Japan; e Health Services Research and Development Center, University of Tsukuba, 1-1-1 Ten-nodai, Tsukuba, Ibaraki, 305-8575, Japan

**Keywords:** Chronic disease, Self-management, Self-efficacy for managing chronic disease scale, Quality of life, Japan

## Abstract

**Background:**

Although accurately assessing self-efficacy for self-management is crucial in chronic illness care, important, there is a scarcity of validated psychometric properties in Japan. This study aimed to validate a Japanese version of the Self-Efficacy for Managing Chronic Disease (SEMCD-J) scale.

**Methods:**

Individuals with self-reported chronic diseases, symptoms, or conditions for over one year were recruited online. The SEMCD-J was translated through translation, back translation, and cognitive interviews with 15 participants. Patient Health Questionnaire-9 (PHQ-9) and Center for Disease Control and Prevention Healthy Days Core Module-4 (CDC-HRQOL-4) were used to evaluate the scale's validity. Test-retest reliability was assessed two weeks after the initial measurement.

**Results:**

Of 500 participants, 494 were analyzed and 149 were analyzed for test-retest reliability. The mean item score was 5.54 ± 2.12. Confirmatory factor analysis (CFA) with a 1-factor model showed good fit (CFI = 0.94, TLI = 0.90, RMSEA = 0.23, SRMR = 0.04). CFA with a 2-factor model showed better fit (CFI = 0.99, TLI = 0.98, RMSEA = 0.11, SRMR = 0.01). Cronbach's α of the total, Factor 1, and Factor 2 were 0.97, 0.97, and 0.92. Their item-total correlations ranged from 0.88 to 0.95, 0.93 to 0.97, and 0.92 to 0.93, respectively. Their interclass correlation coefficients were 0.63, 0.58, and 0.66, respectively. The minimum detectable change was 3.56. Pearson's correlation analyses indicated that SEMCD-J was significantly associated with PHQ-9 (r = −0.57, p < 0.001) and the dimensions of CDC-HRQOL-4, namely, the degree of general health (r = −0.55, p < 0.001), physically unhealthy days (r = −0.46, p < 0.001), mentally unhealthy days (r = −0.48, p < 0.001), and functionally unhealthy days (r = −0.50, p = < 0.001).

**Conclusion:**

The validity and reliability of the SEMCD-J were indicated to be acceptable.

## 1 Introduction

Chronic diseases are diseases, symptoms, and conditions that persist for over a year [1]. Globally, 16–57 % of adults have chronic diseases, inducing problems in multiple domains, such as poor health-related quality of life (QOL), greater mortality, and risk of depression [[2], [3], [4]]. Furthermore, they are associated with relatively high healthcare costs due to increased physician visits, hospitalizations, and medications [5]. Approximately one in four or five individuals with chronic diseases experience difficulties in daily life and social dysfunction [6].

Self-management, a strategy for mitigating symptoms and psychosocial burdens, is essential for the care of people with chronic diseases. Self-management addresses the medical care, social roles, and emotional domains associated with chronic diseases, such as actively participating in care, obtaining social support and information from the environment, and building partnerships with healthcare providers [7]. Its foundation lies in Bandura's theory of self-efficacy, which emphasizes confidence in solving problems and overcoming challenges. This theory is reflected in confidence in the ability to manage problems related to the medical, social, and emotional aspects of chronic diseases [8]. Effective self-management can improve symptom severity [9,10], hospitalization and hospital stay [11], economic cost [12], QOL [13], and social participation in patients with chronic diseases [14].

In Japan, where the proportion of adults aged 65 years or older is 29 % [15], high-quality studies on enhancing patients' self-management skills should be conducted. A few clinical trials of self-management interventions have been performed in Japan [[16], [17], [18], [19]]; however, internationally validated tools that can evaluate all aspects of self-management skills are still lacking. The Self-Efficacy for Managing Chronic Disease (SEMCD) scale [[20], [21], [22], [23]] is a comprehensive tool for self-managing chronic diseases that has been used worldwide. It is based on Bandura's self-efficacy theory and evaluates the core set of self-management behaviors, such as managing physical symptoms and depression, obtaining information and help from others, communicating with physicians and social communities, and others [21]. It has been translated into 11 languages and is one of the gold-standard patient-reported tools for chronic disease self-management [[24], [25], [26], [27], [28], [29], [30], [31], [32], [33], [34], [35], [36], [37]]. Therefore, this study aimed to evaluate the validity and reliability of the Japanese version of the SEMCD (SEMCD-J) in a Japanese population with chronic diseases.

## 2 Materials and methods

### 2.1 SEMCD

The SEMCD contains six items, including those on confidence in the psychological and behavioral management of chronic diseases [20]. Each item is rated on a Likert scale ranging from 1 (not at all confident) to 10 (totally confident). The total score is calculated as the average of the six items; higher average scores indicate greater self-efficacy for managing chronic diseases. The validation study based on multiple cohorts of the original version showed that Cronbach's α was 0.88–0.91 [20]. Correlation with health distress, illness intrusiveness, and activity limitation, were −0.29 to −0.64, −0.47 to 0.62, and −0.33 to −0.54 [20].

### 2.2 Translation process of the self-efficacy for managing chronic disease Japanese version (SEMCD-J)

The SEMCD-J was translated and validated using steps guided by the COnsensus-based Standards for the selection of health Measurement INstruments reporting guidelines [24]. First, the translation from English to Japanese was performed with the permission of the original developers [20]. A healthcare provider (MH) and a non-healthcare provider (MK), both native Japanese speakers, independently translated the SEMCD. Both translations were integrated to create Version 1 through discussion. A researcher proficient in English who resided in an English-speaking region (AN) performed the back-translation without referring to the original version. By addressing the discrepancy between the original and back-translated versions, AN, MK, and MH modified Version 1 to create Version 2.

Second, cognitive interviews were conducted to verify the comprehensibility of the translated SEMCD sentences. We interviewed 15 participants who had diseases, symptoms, or conditions for one year or more and received treatment in February 2024. Participants were recruited through a free online written survey provided by a Japanese Internet survey agency containing approximately 2.2 million active panels in September 2022 in Japan (Rakuten Insight, Tokyo, Japan). Participants were asked to respond to the items of the SEMCD-J Version 2 and indicate phrases or sentences that they found difficult to understand.

As the cognitive interviews identified no phrases or sentences that were difficult to understand, we decided to use Version 2 as the final SEMCD-J version.

### 2.3 Validity and reliability of SEMCD-J

#### 2.3.1 Participants and setting

An online survey was conducted using Rakuten Insight in March 2024. We recruited 500 participants to obtain a sufficient sample size for Confirmatory factor analyses (CFA): both the rules of thumb and the rules based on Monte Carlo simulation studies indicates the model is evaluated safely when the sample is 500 or over [25]. For test-retest reliability and standard error of measurement (SEM), 150 participants re-answered the SEMCD-J two weeks after the initial survey [26]. We decided on the sample size for test-retest reliability based on previous studies recommending a sample size of over 100 participants for precise estimation [27].

We included those who self-reported as 1) 18 years or older and 2) having chronic diseases or conditions with active (medical) treatment for one year or more [1]. Conversely, participants with the following responses were considered to have inappropriate responses and were thus excluded from the analyses: 1) no apparent diseases, symptoms, or conditions, and 2) same rating for all questions because they were considered error responses.

#### 2.3.2 Measurements

##### 2.3.2.1 Patient Health Questionnaire-9 (PHQ-9)

Depression severity was measured using the PHQ-9 [28,29], comprising nine items evaluated using a 4-point Likert scale (0, not at all; 1, several days; 2, more than half the days; 3, nearly every day). The total score ranges from 0 (no depression) to 27 (most severe depression); higher total scores indicate more severe depression. , and the sensitivity and specificity of PHQ-9 ≥ 10 to major depression were 88 % and 88 % in the original version and 90.0 % and 76.6 % in the Japanese version [28,29]. Cronbach's α and correlation of the test-retest reliability in 48 h were 0.89 and 0.84 in the original version [28].

##### 2.3.2.2 Center for Disease Control and Prevention Healthy Days Core Module-4 (CDC-HRQOL-4)

Health-related QOL was assessed using the CDC-HRQOL-4 [30,31]. It comprises 4 items measuring different aspects of chronic disease impact: degree of general health using a 5-point Likert scale (1, excellent; 2, very good; 3, good; 4, fair; 5, poor), physically unhealthy days during the past 30 days, mentally unhealthy days during the past 30 days, and functionally unhealthy days caused by health conditions sduring the past 30 days. Each item was evaluated individually, with higher values indicating more unhealthy conditions. This scale was developed by CDC and used for national survey in the united states, such as Behavioral Risk Factor Surveillance System [32]. Cronbach's α was 0.80 and correlation with SF-8 domains were 0.25–0.67 in the Japanese version [31].

##### 2.3.2.3 Demographic characteristics

The following participant characteristics were collected: sex (male, female, and other), educational attainment (junior high school, high school, junior college or special school, university, and postgraduate), marital status (married or unmarried), presence of children (yes or no), duration of chronic illness (years), duration of medical treatment (years), and type of chronic illness [20] (cardiac disease, respiratory disease, stroke, rheumatic arthritis, diabetes, hypertension, psychiatric disease, chronic fatigue syndrome, multiple sclerosis, low back pain, cancer, migraine, or others).

#### 2.3.3 Statistical analyses

##### 2.3.3.1 Descriptive characteristics

Mean and standard deviation (SD) were calculated for continuous variables, and numbers and proportions were calculated for categorical variables. The median and interquartile ranges (IQR) of the total score and each item on the SEMCD-J were calculated. Shapiro–Francia and kurtosis tests were performed to examine the normality of the SEMCD-J items.

##### 2.3.3.2 Structural validity

CFA with Maximum Likelihood were performed. While most previous studies confirmed the 1-factor model [[33], [34], [35], [36], [37], [38], [39]], a 2-factor model [40,41] separates psychological and behavioral attitudes, was also examined. Therefore, we constructed 1-factor and 2-factor models (Items 1–4 vs. Items 5–6) [40,41] and compared the fit statistics.

The goodness of fit was confirmed using the Tucker–Lewis index (TLI) ≥ 0.90, comparative fit index (CFI) ≥ 0.90, root mean square error of approximation (RMSEA) ≤ 0.06, the Standardized Root Mean Squared Residual (SRMR) ≤ 0.08 [42], Akaike information criterion (AIC), and Bayesian information criterion (BIC). The goodness of fit of the 1-factor and 2-factor models were also compared using the likelihood-ratio test.

##### 2.3.3.3 Criterion validity

Criterion validity was confirmed using Pearson's correlations of the SEMCD-J with the PHQ-9 and each item of the CDC-HRQOL-4 scale. This approach was similar to the original SEMCD validation, which examined its correlation with depression and multiple QOL domains [20].

##### 2.3.3.4 Reliability

Internal consistency was confirmed using Cronbach's alpha and item (I-T) correlations. Cronbach's α was considered desirable when the value was 0.70 or higher [43]. Test-retest reliability was confirmed by calculating the intraclass correlation coefficient (ICC) using time 1 (T1) and time 2 (T2) data. The SEM and minimal detectable change (MDC) were calculated using ICC [44,45]. SEM was calculated using the following formula: SEM = SD × √(1−ICC). MDC was calculated using the following formula: MDC = SEM × 1.96 × √2. A paired *t*-test was performed to compare the SEMCD-J scores at T1 and T2.

All analyses were conducted using Stata 18.0 (College Station, TX, Stata Corp LLC). α was set at 0.05. No missing data were observed.

### 2.4 Ethics

This study was approved by the Research Ethics Committee of the National Center of Neurology and Psychiatry (A2023-134) and in accordance with the Declaration of Helsinki. Electronic informed consent was obtained from all participants through the online survey.

## 3 Results

### 3.1 Characteristics of participants

**Fig. 1:**
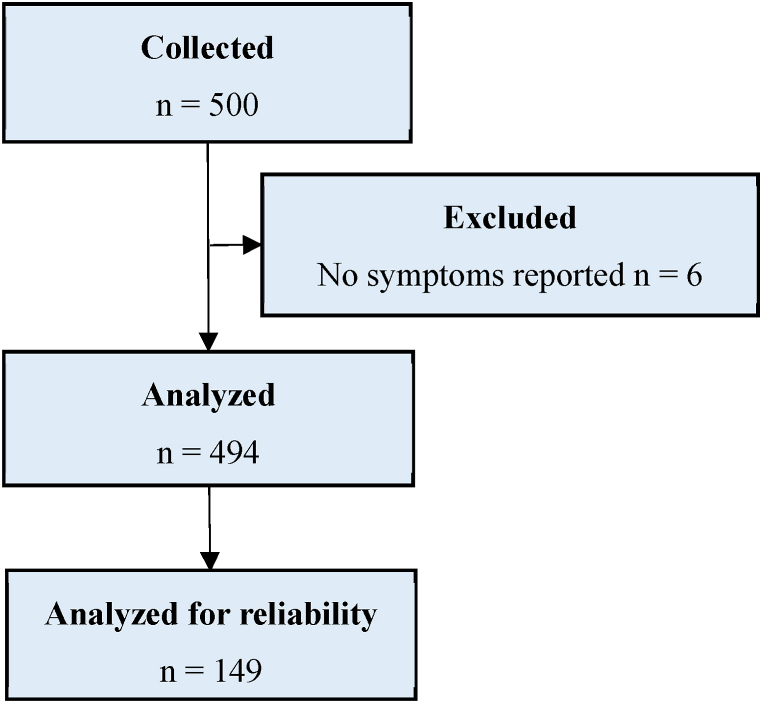
Data collection process.

Of the 500 participants, 496 were analyzed after excluding four who failed to report any specific symptoms or conditions (Fig. 1). No responses reporting the same alternatives were found in the questionnaire.

Table 1 depicts participants’ demographic characteristics. The mean age was 54.81 years (SD = 11.00), and 69.43 % were men. The mean durations of chronic disease and medical treatment were 10.26 years (SD = 8.71) and 9.23 years (SD = 8.20), respectively. The most prevalent chronic disease was hypertension (45.34 %), followed by back pain (24.70 %) and diabetes (16.80 %). The mean PHQ-9 was 6.62 (SD = 6.40). The mean degree of general health was 3.56 ± 1.03. Mean physically, mentally, and functionally unhealthy days were 7.15 (SD = 9.90), 5.69 (SD = 9.75), and 4.51 days (SD = 8.35).

**Table 1:** Demographic characteristics of participants.

	n = 494	
	Mean or n	SD or %
Age	54.81	11.00
Sex		
Male	343	69.43 %
Female	149	30.16 %
Other	2	0.40 %
Educational attainment		
Junior high school	8	1.62 %
High school	125	25.30 %
Junior college or special school	102	20.65 %
University	226	45.75 %
Postgraduate	33	6.68 %
Marital status		
Married	315	63.77 %
Unmarried	179	36.23 %
Status of children		
Yes	284	57.49 %
No	210	42.51 %
Duration of chronic disease (year)	10.26	8.71
Duration of medical treatment (year)	9.23	8.20
Type of chronic diseases		
Hypertension	224	45.34 %
Low back pain	122	24.70 %
Diabetes	83	16.80 %
Psychiatric disease	76	15.38 %
Migraine	65	13.16 %
Respiratory disease	35	7.09 %
Cardiac disease	34	6.88 %
Cancer	17	3.44 %
Rheumatic arthritis	10	2.02 %
Chronic fatigue syndrome	9	1.82 %
Stroke	5	1.01 %
Multiple Sclerosis	0	0.00 %
Others	62	12.55 %
PHQ-9	6.62	6.40
CDC-HRQOL-4		
Degree of general health	3.56	1.03
Excellent	14	2.83 %
Very good	61	12.35 %
good	147	29.76 %
Fair	176	35.63 %
Poor	96	19.43 %
Physically unhealthy days per 30 days	7.15	9.90
Mentally unhealthy days per 30 days	5.69	9.75
Functionally unhealthy days per 30 days	4.51	8.35

PHQ-9, Patient Health Questionnaire-9; CDC-HRQOL-4, Center for Disease Control and Prevention Healthy Days Core Module– 4; SD, Standard Deviation.

Table 2 presents the descriptive characteristics of the SEMCD-J. The mean item scores ranged from 5.31 to 5.70 (SD = 2.22 to 2.36), and the median item scores ranged from 5 to 6 (IQR = 4–7). Kurtosis was significant for all the items (Appendix 1). The mean total score was 5.54 (SD = 2.12), and the median was 5.33 (IQR = 4.17–7.0).

**Table 2:** Characteristics, validity, and reliability of SEMCD-J.

	Mean	SD	Median	IQR	ICC	Factor loading			I-T correlation		
						1-factor model	2-factor model		1-factor model	2-factor model	
Item 1	5.64	2.37	6	4–7	0.54	0.92	0.92		0.93	0.93	
Item 2	5.63	2.28	5	4–7	0.52	0.94	0.94		0.94	0.94	
Item 3	5.63	2.36	5	4–7	0.57	0.96	0.97		0.95	0.97	
Item 4	5.70	2.37	6	4–7	0.58	0.95	0.95		0.95	0.95	
Item 5	5.31	2.22	5	4–7	0.60	0.82		0.93	0.89		0.93
Item 6	5.34	2.23	5	4–7	0.57	0.82		0.92	0.88		0.92
Total	5.54	2.12	5.33	4.17–7	0.63						
Factor 1	5.65	2.24	5.25	4.25–7.25	0.58						
Factor 2	5.32	2.14	5	4–7	0.66						

SD, Standard Deviation; IQR, Interquartile Range; ICC, Intraclass Coefficient; I-T correlation, item-total correlation.

### 3.2 Structural validity

The CFA results indicate that the factor loadings of the 1-factor model ranged from 0.82 to 0.96. CFI, TLI, RMSEA, SRMR, AIC, and BIC were 0.94, 0.90, 0.23, 0.04, 9619.88, and 9695.62, respectively.

The factor loadings of the 2-factor model ranged from 0.93 to 0.97 for Factor 1 and from 0.92 to 0.93 for Factor 2. CFI, TLI, RMSEA, SRMR, AIC, and BIC were 0.99, 0.98, 0.11, 0.01, 9436.96, and 9516.80, respectively. The likelihood-ratio test indicated that the 2-factor model had better fit than the 1-factor model (χ2 = 185.02, p < 0.001).

No issues, such as convergence problems, improper estimation, or model identification difficulties, were observed.

### 3.3 Reliability

Cronbach's α of SEMCD-J total, Factor 1, and Factor 2 were 0.97, 0.97, and 0.92, respectively. The I-T correlations ranged from 0.88 to 0.95. The ICC of the SEMCD-J total score and Factors 1 and 2 were 0.63, 0.58, and 0.66, respectively. The ICC of the items ranged from 0.52 to 0.60. The SEM of the SEMCD-J total score and Factors 1 and 2 were 1.28, 1.45, and 1.26, respectively. Their MDCs were 3.56, 4.03, and 3.48, respectively.

### 3.4 Criterion validity

As Table 3 shows, SEMCD-J was significantly correlated with PHQ-9 (r = −0.57, p < 0.001), degree of general health (r = −0.56, p < 0.001), physically unhealthy days (r = −0.45, p < 0.001), mentally unhealthy days (r = −0.49, p < 0.001), and functionally unhealthy days (r = −0.51, p < 0.001).

**Table 3:** The criterion validity of SEMCD-J.

	r	p
PHQ-9	−0.57	<0.001
CDC-HRQOL-4		
Degree of general health	−0.55	<0.001
Physically unhealthy days per 30 days	−0.46	<0.001
Mentally unhealthy days per 30 days	−0.48	<0.001
Functionally unhealthy days per 30 days	−0.50	<0.001

PHQ-9, Patient Health Questionnaire-9; CDC-HRQOL-4, Center for Disease Control and Prevention Healthy Days Core Module– 4.

## 4 Discussion

In this study, we examined the validity and reliability of a Japanese version of the SEMCD. The structural validity, internal consistency, and criterion validity were indicated to be sufficient, although partial structural validity, including RMSEA, and test-retest reliability, was not indicated to be excellent. This is the first study to validate the SEMCD in Japan using an appropriate procedure, sufficient sample size, and participant variation. The SEMCD-J can be adapted for multiple chronic diseases in Japan.

Fit indices, including CFI, TLI, and SRMR, indicated the structural validity was sufficient with the 1-factor model as with English and French versions [23,46], although RMSEA was not sufficient. This suggestion can be interpreted to mean that it is reasonable to use the scale as a 1-factor structure. Furthermore, the fit indices for the 2-factor model were sufficient, similar to those of the Chinese and Turkish versions [40,41]. This model may be inevitable because each factor appears to be divided into the two original scales on which the SEMCD was based, except for Item 3 [20,22]. According to the Chinese and Turkish versions [40,41], Factors 1 and 2 measure psychological and behavioral attitudes, respectively. In addition to the total scale, subscales can also be utilized. The two-factor solution may provide more detailed assessment, distinguishing between general management skills and emotional coping abilities, which require different approaches: behavioral and cognitive-emotional strategies, respectively.

Considering kurtosis by midpoint responding in this data and the tendency for RMSEA to be influenced by the normality of data [42], CFI, TLI, and SRMR might provide a more accurate evaluation in this study. Generally, midpoint responses were frequently observed when the respondents did not have sufficient knowledge or understanding to answer questions about what was being asked in the items [47]. Thus, midpoint responses in this study may stem from a lack of knowledge or understanding of self-management of chronic diseases in some participants. The online survey further challenges the uniformity of the participants’ understanding and knowledge of self-management and the quality of the medical institutions they visit. Conversely, participants in previous studies, who visited institutions focused on self-management enough to validate SEMCD versions, may have displayed a relatively high understanding of self-management. The understandability of the SEMCD-J wording is ensured based on an accurate translation procedure and cognitive interviews.

Criterion validity was indicated to be sufficient for moderate associations between the SEMCD-J and depression and QOL, consistent with previous studies [20,23,34,37,39,48,49]. The association between SEMCD-J and depression can be accounted for by the following process: depression is suggested to inhibit adherence to self-management, resulting in relatively low scores of SEMCD-J among depressive participants, based on studies indicating associations between depression and adherence to self-management among several chronic diseases such as diabetes, hypertension, dyslipidemia, and chronic pain [[50], [51], [52]]. Based on a systematic review indicating that interventions promoting self-management enhance QOL among individuals with chronic diseases, higher QOL can be induced by higher self-management self-efficacy [13].

Regarding reliability, the internal consistency was interpreted as sufficient, although Cronbach's α is limited in appropriateness as the marker of internal consistency [53,54]. The test-retest reliability was acceptable but lower than that in other studies, despite the interval being similar or shorter in this study [34,36,40,41,48,55,56]. The online survey allowed for anonymous responses, making it easier for participants to answer honestly without worrying about others' opinions [57]. Consistent self-management engagement requires active effort [58], which is especially challenging for individuals with hypertension and diabetes [50], which were common diseases among the participants in this study; thus, participants' honest responses, a situation that in fact does not always allow for stable self-management, might have affected test-retest reliability. Instead, we calculated MDC. The score of SEMCD-J should be interpreted as genuine change when 3.56 or over change was observed. This calculation helps to complement the reliability assessment and ensures that actual change are accurately captured.

This study had some limitations. First, our findings may be limited to individuals with chronic diseases whose symptoms were mild to moderate enough to participate in the study. Second, the diagnostic accuracy in this study was not as high as that of participants collected from medical institutions. The results reflect the characteristics of hypertension more adeptly than those of other chronic diseases because approximately half of the sample had hypertension. Third, high Cronbach's α values and factor loadings might be due to participants rating all items with the same value, making their responses redundant. Forth, the comprehensibility of factor 2 might be limited due to containing only two items. However, it has been reported that sample size of over 400 is reported to be sufficient for analyzing a factor with two items [25], thus believed to avoid unstable validity and reliability. Finally, although other fit indices and factor loadings were acceptable, the RMSEA values were insufficient. We interpret this as likely due to data distribution rather than model issues, given this study's confirmatory nature based on the original scale model.

## 5 Conclusion

In this study, we examined the validity and reliability of the SEMCD-J for individuals with chronic diseases. The SEMCD-J is indicated to have sufficient criterion and structural validity, internal consistency, and acceptable test-retest reliability. Further studies to examine test-retest reliability and structural validity with different Japanese samples should be performed to confirm whether these findings are specific to Japanese people with chronic disorders.

## CRediT authorship contribution statement

**Megumi Hazumi:** Writing – review & editing, Writing – original draft, Validation, Project administration, Methodology, Investigation, Funding acquisition, Formal analysis, Data curation. **Mayumi Kataoka:** Writing – review & editing, Methodology, Investigation, Funding acquisition, Conceptualization. **Ayako Nakashita:** Writing – review & editing, Methodology, Investigation. **Kentaro Usuda:** Writing – review & editing, Funding acquisition. **Michi Miyake:** Writing – review & editing. **Chiaki Kamikawa:** Writing – review & editing. **Daisuke Nishi:** Writing – review & editing, Funding acquisition. **Naoaki Kuroda:** Writing – review & editing.

## Data availability

The data are not available due to the limitations of the ethical approval from the Research Ethics Committee of the National Center of Neurology and Psychiatry (A2023-134), not including provisions for data sharing beyond the scope of the current study and the NCNP institute.

## Funding

This study was supported by an Intramural Research Grant for Neurological and Psychiatric Disorders from the 10.13039/501100009438NCNP (4-3)

## Declaration of competing interest

The authors declare the following financial interests/personal relationships which may be considered as potential competing interests:Daisuke Nishi reports personal fees outside of the submitted work from Startia, Inc., en-Power, Inc., MD.net, and 10.13039/100008373Takeda Pharmaceutical Company, Ltd. If there are other authors, they declare that they have no known competing financial interests or personal relationships that could have appeared to influence the work reported in this paper.

## Figures and Tables

**Figure:**
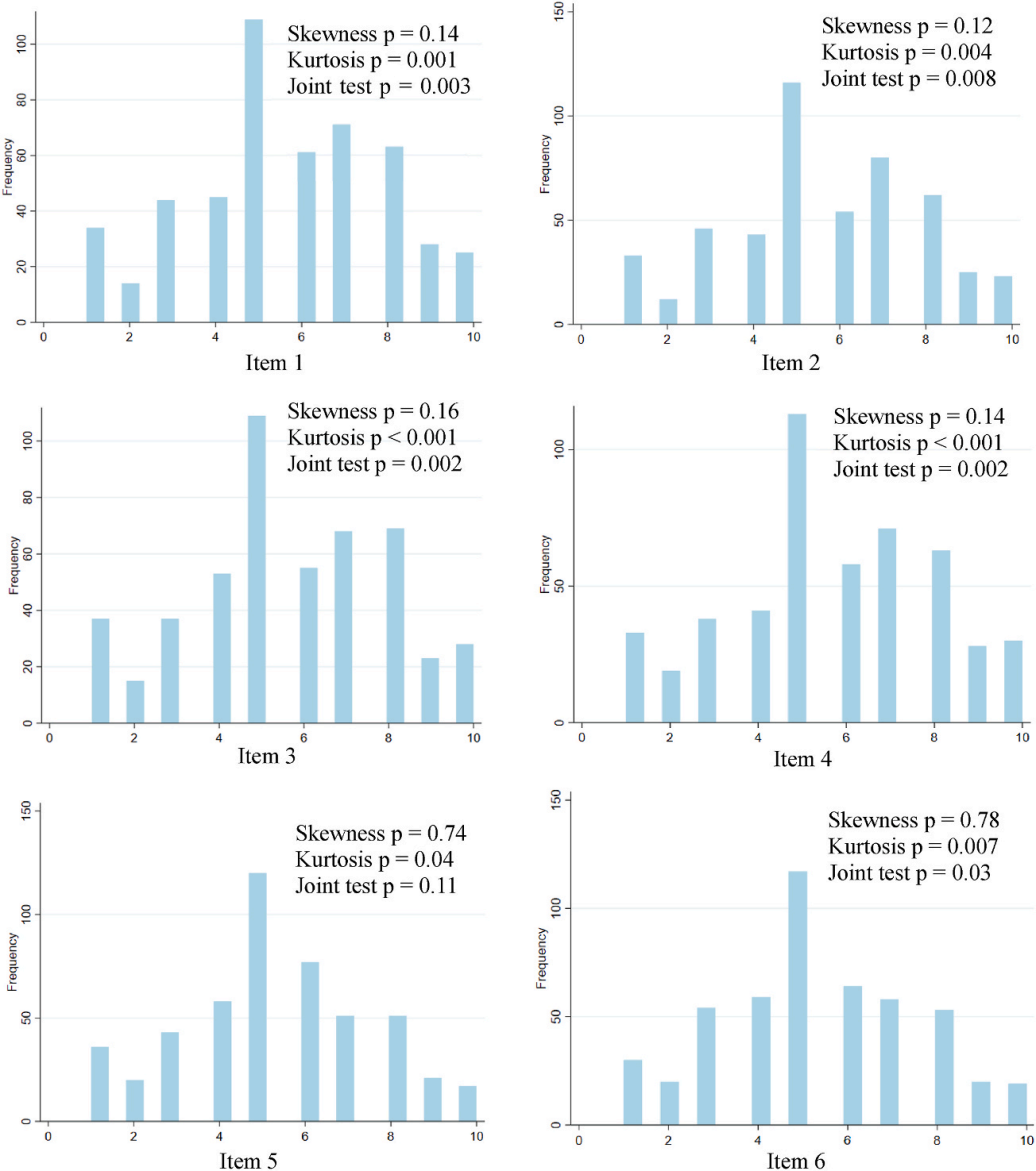

